# Chylomicron Retention Disease: Failure to Thrive and Abdominal Distention in an Infant

**DOI:** 10.1097/PG9.0000000000000145

**Published:** 2021-11-29

**Authors:** Krisha Nayak, Judy Fuentebella

**Affiliations:** From the * Pediatrics, Kaiser Permanente, Oakland, CA; † Pediatric Gastroenterology, Kaiser Permanente, Oakland, CA.

**Keywords:** failure to thrive, abdominal distention, lipid malabsorption disorder, lipid-laden enterocytes, hypocholesterolemia

## Abstract

This case report describes an infant with failure to thrive and progressive abdominal distention that ultimately led to a rare diagnosis of chylomicron retention disease at 1 year of life. Laboratory abnormalities included increased qualitative stool fat, along with low apolipoprotein B, high-density lipoprotein, low-density lipoprotein (LDL), and total cholesterol in blood. In chylomicron retention disease, diarrhea has been reported as the most common presenting symptom followed by failure to thrive and vomiting. Diarrhea and vomiting before 6 months of life have been described in cases of chylomicron retention disease reported in the literature; however, this patient did not present with either of those symptoms. This case report uniquely demonstrates that lack of early or persistent digestive symptoms of diarrhea or vomiting does not exclude a diagnosis of chylomicron retention disease.

## INTRODUCTION

Chylomicron retention disease (CRD) is a very rare, fat malabsorption disorder that typically presents in infancy. Manifestations of this disease include diarrhea, failure to thrive, vomiting, and abdominal distention. There have been no reported cases of CRD without either chronic diarrhea or frequent vomiting.

## CASE REPORT

An ex-term 5-month-old male presented to the Gastroenterology clinic due to failure to thrive despite feeding well on fortified feeds of breast milk. In the neonatal period, the only issues identified were failure to surpass birth weight (3.38 kg) by 4 weeks of life and a history of spit ups after each feed. There was no history of vomiting. He had 2–3 mushy stools per day without blood, mucous, or oil droplets. Ranitidine was initiated for a presumptive diagnosis of GERD, and the patient was transitioned to nondairy breast milk and hypoallergenic formula for possible milk protein allergy. However, the patient continued to fall further from the growth curve. By the time of referral to Pediatric Gastroenterology, his weight was 4.41 kg and weight-for-age was below the 0.01th percentile.

Evaluation before his Gastroenterology clinic visit included normal BMP, CBC, urinalysis, and abdominal radiograph. Abdominal ultrasound did not show any abnormal masses, ascites, or organomegaly. Other labs obtained showed ALT 67 U/L (slight elevation), A tocopherol 1.4 mg/L, beta gamma tocopherol <1.0 mg/L, total protein 5.4 g/dL, and albumin 3.9 g/dL. Stool studies were significant for increased qualitative stool fat (single sample) with normal neutral fat and normal elastase.

The patient was eventually admitted to the hospital at 7 months of age for failure to thrive and progressive abdominal distention. Further evaluation revealed normal stool alpha 1 antitrypsin, stool reducing substance, sweat test, TTG IgA, total IgA, and DGP IgA. Repeat qualitative stool fat (single sample) was positive. Stool calprotectin was 134 µg/g. He had mildly elevated ALT of 99 U/L and AST of 91 U/L. Nasogastric tube feeds were started, and formula was changed to Elecare.

One week after discharge, he was able to adequately gain weight and meet his caloric goal via oral feeds alone. However, the infant continued to have significant abdominal distention. Barium enema was normal. His ALT and AST peaked at 260 U/L and 121 U/L, respectively. GGT, total bilirubin, and direct bilirubin remained normal. Furthermore, the patient was unable to progress to solid foods. The occupational therapist assessed him to have oral motor incoordination and oral sensory aversion to food. An upper endoscopy was scheduled to evaluate his feeding issues and poor weight gain. A flexible sigmoidoscopy and rectal suction biopsy were also planned.

**FIGURE 1.**
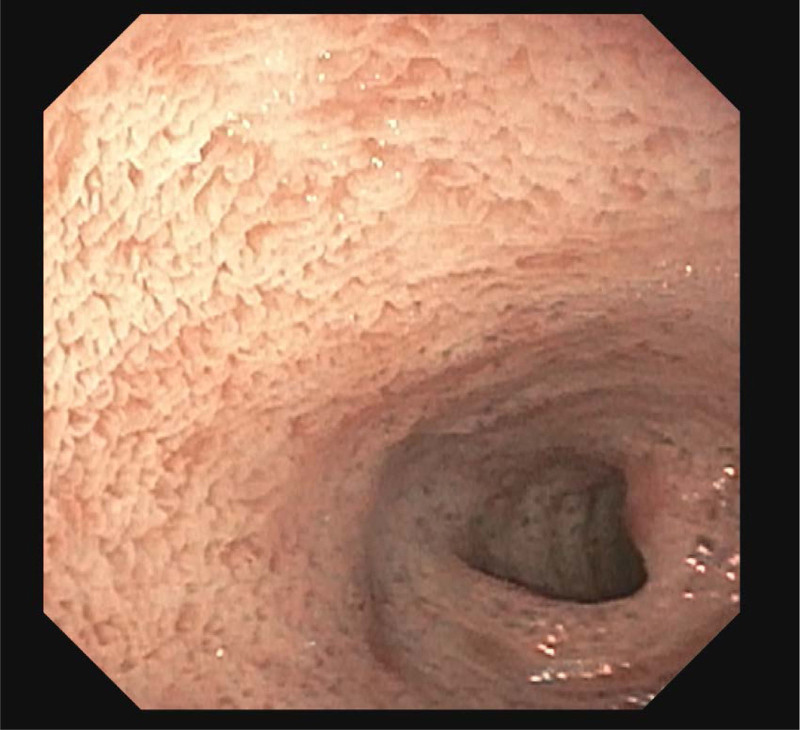
Whitish appearing duodenal mucosa with normal villi.

His colonic biopsies were normal and rectal suction biopsy showed ganglion cells, which ruled out Hirschsprung’s disease. On upper endoscopy, whitish appearing duodenal mucosa with normal villi was visualized (Fig. 1). Duodenal biopsies showed increased intraepithelial lipid accumulation, which was suggestive of abetalipoproteinemia. However, parents had both tested negative for this condition. Further evaluation revealed low apolipoprotein B (30 mg/dL), low high-density lipoprotein (21 mg/dL), low LDL (26 mg/dL), low total cholesterol (52 mg/dL), normal triglyceride (60 mg/dL), and normal levels of vitamins A, D, and E. Genetic testing revealed two pathogenic variants in SAR1B: a maternally inherited SAR1B c.247 C>T (p.Arg83Ter) and a de novo SAR1B c.409 G>A (p.Asp137Asn).

The patient was diagnosed with chylomicron retention disease at 12 months of age. His formula was changed to low-fat Vivonex. Bowel movements occurred 1–2 times per day and were more formed. His transaminases trended downwards. His abdominal distention resolved and his weight increased from the 2nd to the 63rd weight-for-age percentile over 4 months.

## CASE DISCUSSION

Chylomicron retention disease is a recessively inherited, rare lipid malabsorption disorder that results from mutations in SARA2 and SAR1B genes (1,2). These genes encode for the SAR1B GTPase protein, which is necessary for the transport of prechylomicron vesicles from the endoplasmic reticulum to the golgi apparatus and for the fusion of these vesicles to the golgi apparatus. In CRD, hypocholesterolemia occurs because the pre-chylomicron transport vesicles lack the ability to be exported out of the enterocytes (2–4). Due to this defective mechanism, endoscopy of patients with CRD reveals white duodenal and jejunal mucosa with lipid-laden enterocytes on biopsy (2,3).

In patients with CRD, the onset of malabsorption symptoms has been reported to occur within the first 6 months of life. The primary symptoms previously described in CRD include diarrhea, failure to thrive, vomiting, and abdominal distention, with diarrhea being the main clinical manifestation followed by failure to thrive (3). In fact, diarrhea has been reported in all cases of CRD except for one case report of an infant who presented with vomiting and failure to thrive (3,5). Absence of diarrhea is atypical in fat malabsorption. The patient originally presented with failure to thrive and progressive abdominal distention, but he had normal bowel movements and no vomiting.

This patient also exhibited inability to progress to solid foods, hypotonia, and delay in developmental milestones. Of note, creatine kinase was normal at 111 U/L. On developmental testing at 17–19 months of age, he demonstrated delays in expressive language and gross motor skills. His inability to progress to solid foods may have been secondary to fat intolerance or abnormal oral-motor coordination for age. Neurological manifestations such as hyporeflexia, areflexia, myopathy, and visual abnormalities have been described in CRD; however, they commonly present in late childhood or adulthood (1,3).

To make a definitive diagnosis of CRD, endoscopy with biopsy and genetic testing to identify pathogenic variants in SAR1B and SARA2 are needed. The abnormal lipid profile seen in CRD can help to differentiate it from other genetic hypocholesterolemias, including abetalipoproteinemia (ABL) and homozygous hypobetalipoproteinemia (HBL), which present with significantly decreased triglyceride levels with no measurable LDL levels (3). If a patient presents with the classic abnormal lipid profile with positive stool fat, a clinician should keep CRD on their differential even in the absence of chronic diarrhea or vomiting.
